# Supplementary figures and images for: Bidirectional association between breast cancer and dementia: a systematic review and meta-analysis of observational studies
Source: PeerJ. 2025 Jan 31;13:e18888. doi: 10.7717/peerj.18888 (PMC11789662; doi:10.7717/peerj.18888)

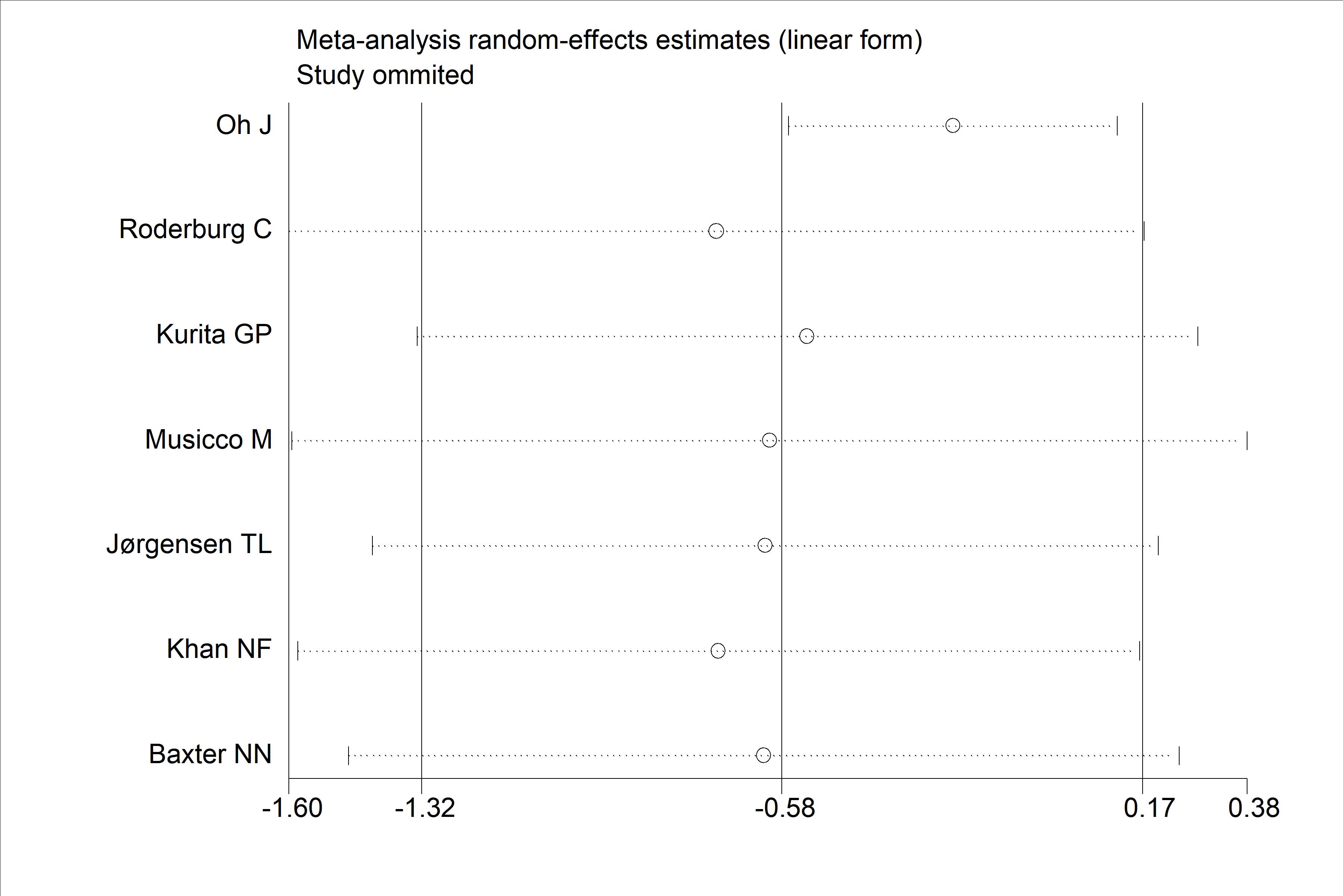

Supplement: Supplemental Information 2 [file peerj-13-18888-s002.jpg]

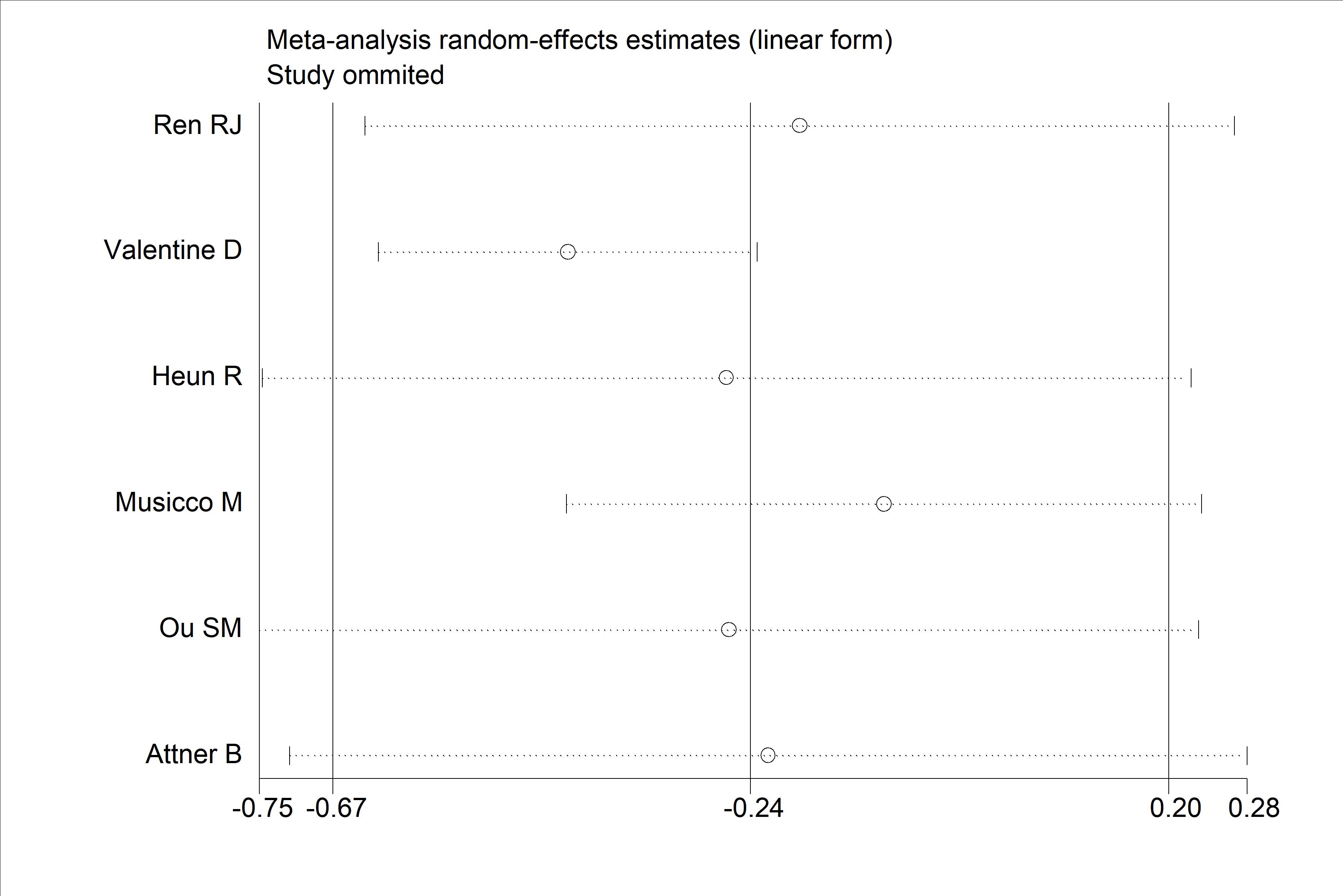

Supplement: Supplemental Information 3 [file peerj-13-18888-s003.jpg]

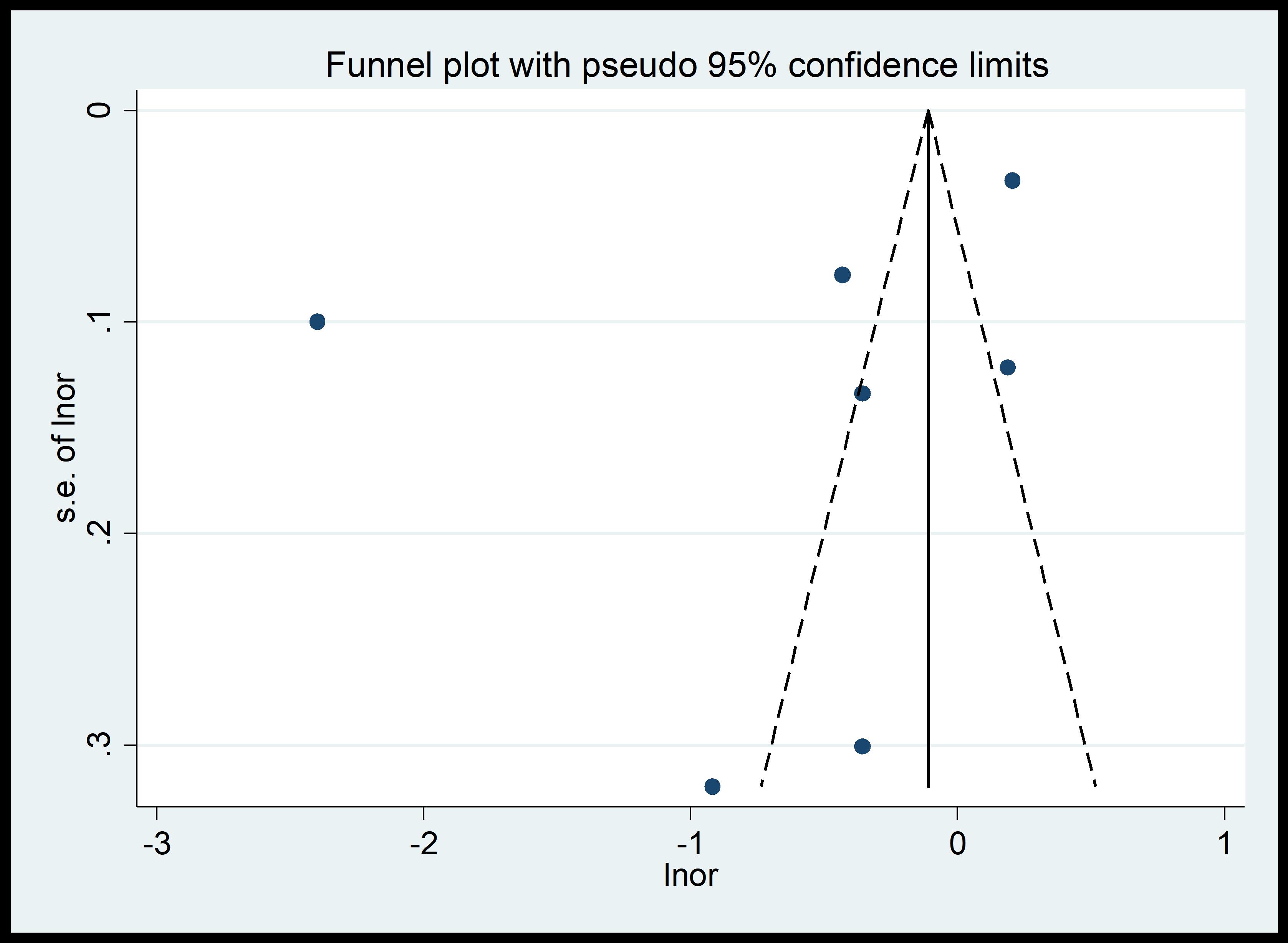

Supplement: Supplemental Information 4 [file peerj-13-18888-s004.jpg]

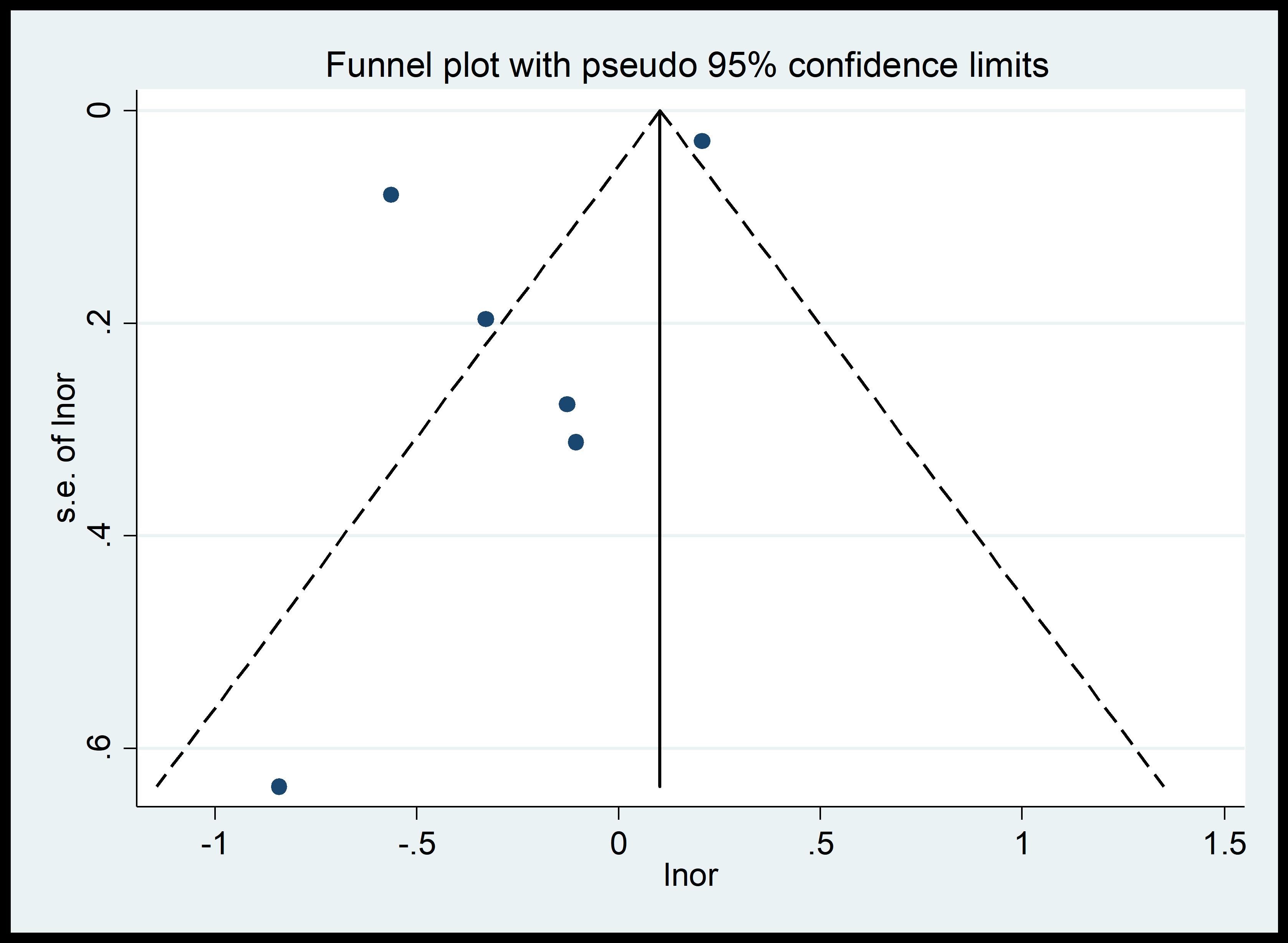

Supplement: Supplemental Information 5 [file peerj-13-18888-s005.jpg]
