# Supplementary material for: Bidirectional association between breast cancer and dementia: a systematic review and meta-analysis of observational studies
Source: PeerJ. 2025 Jan 31;13:e18888. doi: 10.7717/peerj.18888 (PMC11789662; doi:10.7717/peerj.18888)
Supplement: Supplemental Information 6 [file peerj-13-18888-s006.docx]

**Supplemental Table 1** Details of the Literature Search Strategy in PubMed (June 20, 2024)

| **Search** | **Query** | **Results** |
| --- | --- | --- |
| 1 | ((((((((((((((((((((((((((((((((((((((Breast Neoplasms)[MeSH Terms]) OR ((Breast Neoplasm[Title/Abstract]))) OR ((Neoplasm, Breast[Title/Abstract]))) OR ((Neoplasms, Breast[Title/Abstract]))) OR ((Breast Tumors[Title/Abstract]))) OR ((Breast Tumor[Title/Abstract]))) OR ((Tumor, Breast[Title/Abstract]))) OR ((Tumors, Breast[Title/Abstract]))) OR ((Breast Cancer[Title/Abstract]))) OR ((Cancer, Breast[Title/Abstract]))) OR ((Cancer of Breast[Title/Abstract]))) OR ((Cancer of the Breast[Title/Abstract]))) OR ((Malignant Neoplasm of Breast[Title/Abstract]))) OR ((Breast Malignant Neoplasm[Title/Abstract]))) OR ((Breast Malignant Neoplasms[Title/Abstract]))) OR ((Malignant Tumor of Breast[Title/Abstract]))) OR ((Breast Malignant Tumor[Title/Abstract]))) OR ((Breast Malignant Tumors[Title/Abstract]))) OR ((Mammary Cancer[Title/Abstract]))) OR ((Cancer, Mammary[Title/Abstract]))) OR ((Cancers, Mammary[Title/Abstract]))) OR ((Mammary Cancers[Title/Abstract]))) OR ((Mammary Neoplasms, Human[Title/Abstract]))) OR ((Human Mammary Neoplasm[Title/Abstract]))) OR ((Human Mammary Neoplasms[Title/Abstract]))) OR ((Neoplasm, Human Mammary[Title/Abstract]))) OR ((Neoplasms, Human Mammary[Title/Abstract]))) OR ((Mammary Neoplasm, Human[Title/Abstract]))) OR ((Breast Carcinoma[Title/Abstract]))) OR ((Breast Carcinomas[Title/Abstract]))) OR ((Carcinoma, Breast[Title/Abstract]))) OR ((Carcinomas, Breast[Title/Abstract]))) OR ((Mammary Carcinoma, Human[Title/Abstract]))) OR ((Carcinoma, Human Mammary[Title/Abstract]))) OR ((Carcinomas, Human Mammary[Title/Abstract]))) OR ((Human Mammary Carcinomas[Title/Abstract]))) OR ((Mammary Carcinomas, Human[Title/Abstract]))) OR ((Human Mammary Carcinoma[Title/Abstract])) | 505,373 |
| 2 | ((((((((((((Dementia[MeSH Terms]) OR (Dementias[Title/Abstract])) OR (Amentia[Title/Abstract])) OR (Amentias[Title/Abstract])) OR ((Senile Paranoid Dementia[Title/Abstract]))) OR ((Dementias, Senile Paranoid[Title/Abstract]))) OR (Paranoid Dementia, Senile[Title/Abstract]))) OR ((Paranoid Dementias, Senile[Title/Abstract]))) OR ((Senile Paranoid Dementias[Title/Abstract]))) OR ((Familial Dementia[Title/Abstract]))) OR ((Dementia, Familial[Title/Abstract]))) OR ((Dementias, Familial[Title/Abstract]))) OR ((Familial Dementias[Title/Abstract])) | 218,228 |
| 3 | (((((((((((((((((((((((((((((((((((((((Breast Neoplasms)[MeSH Terms]) OR ((Breast Neoplasm[Title/Abstract]))) OR ((Neoplasm, Breast[Title/Abstract]))) OR ((Neoplasms, Breast[Title/Abstract]))) OR ((Breast Tumors[Title/Abstract]))) OR ((Breast Tumor[Title/Abstract]))) OR ((Tumor, Breast[Title/Abstract]))) OR ((Tumors, Breast[Title/Abstract]))) OR ((Breast Cancer[Title/Abstract]))) OR ((Cancer, Breast[Title/Abstract]))) OR ((Cancer of Breast[Title/Abstract]))) OR ((Cancer of the Breast[Title/Abstract]))) OR ((Malignant Neoplasm of Breast[Title/Abstract]))) OR ((Breast Malignant Neoplasm[Title/Abstract]))) OR ((Breast Malignant Neoplasms[Title/Abstract]))) OR ((Malignant Tumor of Breast[Title/Abstract]))) OR ((Breast Malignant Tumor[Title/Abstract]))) OR ((Breast Malignant Tumors[Title/Abstract]))) OR ((Mammary Cancer[Title/Abstract]))) OR ((Cancer, Mammary[Title/Abstract]))) OR ((Cancers, Mammary[Title/Abstract]))) OR ((Mammary Cancers[Title/Abstract]))) OR ((Mammary Neoplasms, Human[Title/Abstract]))) OR ((Human Mammary Neoplasm[Title/Abstract]))) OR ((Human Mammary Neoplasms[Title/Abstract]))) OR ((Neoplasm, Human Mammary[Title/Abstract]))) OR ((Neoplasms, Human Mammary[Title/Abstract]))) OR ((Mammary Neoplasm, Human[Title/Abstract]))) OR ((Breast Carcinoma[Title/Abstract]))) OR ((Breast Carcinomas[Title/Abstract]))) OR ((Carcinoma, Breast[Title/Abstract]))) OR ((Carcinomas, Breast[Title/Abstract]))) OR ((Mammary Carcinoma, Human[Title/Abstract]))) OR ((Carcinoma, Human Mammary[Title/Abstract]))) OR ((Carcinomas, Human Mammary[Title/Abstract]))) OR ((Human Mammary Carcinomas[Title/Abstract]))) OR ((Mammary Carcinomas, Human[Title/Abstract]))) OR ((Human Mammary Carcinoma[Title/Abstract]))) AND ((((((((((((((Dementia[MeSH Terms]) OR (Dementias[Title/Abstract])) OR (Amentia[Title/Abstract])) OR (Amentias[Title/Abstract])) OR ((Senile Paranoid Dementia[Title/Abstract]))) OR ((Dementias, Senile Paranoid[Title/Abstract]))) OR (Paranoid Dementia, Senile[Title/Abstract]))) OR ((Paranoid Dementias, Senile[Title/Abstract]))) OR ((Senile Paranoid Dementias[Title/Abstract]))) OR ((Familial Dementia[Title/Abstract]))) OR ((Dementia, Familial[Title/Abstract]))) OR ((Dementias, Familial[Title/Abstract]))) OR ((Familial Dementias[Title/Abstract]))) AND (((((((((((((Dementia[MeSH Terms]) OR (Dementias[Title/Abstract])) OR (Amentia[Title/Abstract])) OR (Amentias[Title/Abstract])) OR ((Senile Paranoid Dementia[Title/Abstract]))) OR ((Dementias, Senile Paranoid[Title/Abstract]))) OR (Paranoid Dementia, Senile[Title/Abstract]))) OR ((Paranoid Dementias, Senile[Title/Abstract]))) OR ((Senile Paranoid Dementias[Title/Abstract]))) OR ((Familial Dementia[Title/Abstract]))) OR ((Dementia, Familial[Title/Abstract]))) OR ((Dementias, Familial[Title/Abstract]))) OR ((Familial Dementias[Title/Abstract])))) | 422 |
