# Supplementary material for: Bidirectional association between breast cancer and dementia: a systematic review and meta-analysis of observational studies
Source: PeerJ. 2025 Jan 31;13:e18888. doi: 10.7717/peerj.18888 (PMC11789662; doi:10.7717/peerj.18888)
Supplement: Supplemental Information 7 [file peerj-13-18888-s007.docx]

**Supplemental Table 2** Details of the Literature Search Strategy in Cochrane Library (June 20, 2024)

| **Search** | **Query** | **Results** |
| --- | --- | --- |
| #1 | MeSH descriptor:[Breast Neoplasms] explode all trees | 20,230 |
| #2 | (Breast Neoplasm):ti,ab,kw | 7,019 |
| #3 | (Neoplasm, Breast):ti,ab,kw | 7,019 |
| #4 | (Neoplasms, Breast):ti,ab,kw | 22,970 |
| #5 | (Breast Tumors):ti,ab,kw | 5,211 |
| #6 | (Breast Tumor):ti,ab,kw | 11,635 |
| #7 | (Tumor, Breast):ti,ab,kw | 11,635 |
| #8 | (Tumors, Breast):ti,ab,kw | 5,211 |
| #9 | (Breast Cancer):ti,ab,kw | 45,649 |
| #10 | (Cancer, Breast):ti,ab,kw | 45,649 |
| #11 | (Cancer of Breast):ti,ab,kw | 43,644 |
| #12 | (Cancer of the Breast):ti,ab,kw | 40,585 |
| #13 | (Malignant Neoplasm of Breast):ti,ab,kw | 1,452 |
| #14 | (Breast Malignant Neoplasm):ti,ab,kw | 1,471 |
| #15 | (Breast Malignant Neoplasms):ti,ab,kw | 969 |
| #16 | (Malignant Tumor of Breast):ti,ab,kw | 958 |
| #17 | (Breast Malignant Tumor):ti,ab,kw | 960 |
| #18 | (Breast Malignant Tumors):ti,ab,kw | 573 |
| #19 | (Mammary Cancer):ti,ab,kw | 620 |
| #20 | (Cancer, Mammary):ti,ab,kw | 620 |
| #21 | (Cancers, Mammary):ti,ab,kw | 64 |
| #22 | (Mammary Cancers):ti,ab,kw | 64 |
| #23 | (Mammary Neoplasms, Human):ti,ab,kw | 176 |
| #24 | (Human Mammary Neoplasm):ti,ab,kw | 57 |
| #25 | (Human Mammary Neoplasms):ti,ab,kw | 176 |
| #26 | (Neoplasm, Human Mammary):ti,ab,kw | 57 |
| #27 | (Neoplasms, Human Mammary):ti,ab,kw | 176 |
| #28 | (Mammary Neoplasm, Human):ti,ab,kw | 57 |
| #29 | (Breast Carcinoma):ti,ab,kw | 4,659 |
| #30 | (Breast Carcinomas):ti,ab,kw | 296 |
| #31 | (Carcinoma, Breast):ti,ab,kw | 4,659 |
| #32 | (Carcinomas, Breast):ti,ab,kw | 296 |
| #33 | (Mammary Carcinoma, Human):ti,ab,kw | 70 |
| #34 | (Carcinoma, Human Mammary):ti,ab,kw | 70 |
| #35 | (Carcinomas, Human Mammary):ti,ab,kw | 8 |
| #36 | (Human Mammary Carcinomas):ti,ab,kw | 8 |
| #37 | (Mammary Carcinomas, Human):ti,ab,kw | 9 |
| #38 | (Human Mammary Carcinoma):ti,ab,kw | 70 |
| #39 | #1 OR #2 OR #3 OR #4 OR #5 OR #6 OR #7 OR #8 OR #9 OR #10 OR #11 OR #12 OR #13 OR #14 OR #15 OR #16 OR #17 OR #18 OR #19 OR #20 OR #21 OR #22 OR #23 OR #24 OR #25 OR #26 OR #27 OR #28 OR #29 OR #30 OR #31 OR #32 OR #33 OR #34 OR #35 OR #36 OR #37 OR #38 | 48,092 |
| #40 | MeSH descriptor. [Dementia] explode all trees | 9,444 |
| #41 | (Dementias):ti,ab,kw | 591 |
| #42 | (Amentia):ti,ab,kw | 1 |
| #43 | (Amentias):ti,ab,kw | 0 |
| #44 | (Senile Paranoid Dementia):ti,ab,kw | 5 |
| #45 | (Dementias, Senile Paranoid):ti,ab,kw | 0 |
| #46 | (Paranoid Dementia, Senile):ti,ab,kw | 5 |
| #47 | (Paranoid Dementias, Senile):ti,ab,kw | 0 |
| #48 | (Senile Paranoid Dementias):ti,ab,kw | 0 |
| #49 | (Familial Dementia):ti,ab,kw | 40 |
| #50 | (Dementia, Familial):ti,ab,kw | 40 |
| #51 | (Dementias, Familial):ti,ab,kw | 4 |
| #52 | (Familial Dementias):ti,ab,kw | 4 |
| #53 | #40 OR #41 OR #42 OR #43 OR #44 OR #45 OR #46 OR #47 OR #48 OR #49 OR #50 OR #51 OR #52 | 9,817 |
| #54 | #39 AND #53 | 21 |
