# Supplementary material for: Bidirectional association between breast cancer and dementia: a systematic review and meta-analysis of observational studies
Source: PeerJ. 2025 Jan 31;13:e18888. doi: 10.7717/peerj.18888 (PMC11789662; doi:10.7717/peerj.18888)
Supplement: Supplemental Information 8 [file peerj-13-18888-s008.docx]

**Supplemental Table 3** Details of the Literature Search Strategy in Embase (June 20, 2024)

| **Search** | **Query** | **Items found** |
| --- | --- | --- |
| #1 | 'Breast Neoplasms'/exp | 712,623 |
| #2 | 'Breast Neoplasm':ab,ti | 518 |
| #3 | 'Neoplasm, Breast':ab,ti | 30 |
| #4 | 'Neoplasms, Breast':ab,ti | 63 |
| #5 | 'Breast Tumors':ab,ti | 19,204 |
| #6 | 'Breast Tumor':ab,ti | 17,687 |
| #7 | 'Tumor, Breast':ab,ti | 485 |
| #8 | 'Tumors, Breast':ab,ti | 518 |
| #9 | 'ABreast Cancer':ab,ti | 1 |
| #10 | 'Cancer, Breast':ab,ti | 4,652 |
| #11 | 'Cancer of Breast':ab,ti | 175 |
| #12 | 'Cancer of the Breast':ab,ti | 3,040 |
| #13 | 'Malignant Neoplasm of Breast':ab,ti | 34 |
| #14 | 'Breast Malignant Neoplasm':ab,ti | 10 |
| #15 | 'Breast Malignant Neoplasms':ab,ti | 9 |
| #16 | 'Malignant Tumor of Breast':ab,ti | 3 |
| #17 | 'Breast Malignant Tumor':ab,ti | 31 |
| #18 | 'Breast Malignant Tumors':ab,ti | 47 |
| #19 | 'Mammary Cancer':ab,ti | 4,392 |
| #20 | 'Cancer, Mammary':ab,ti | 95 |
| #21 | 'Cancers, Mammary':ab,ti | 95 |
| #22 | 'Mammary Cancers':ab,ti | 589 |
| #23 | 'Mammary Neoplasms, Human':ab,ti | 0 |
| #24 | 'Human Mammary Neoplasm':ab,ti | 0 |
| #25 | 'Human Mammary Neoplasms':ab,ti | 3 |
| #26 | 'Neoplasm, Human Mammary':ab,ti | 0 |
| #27 | 'Neoplasms, Human Mammary':ab,ti | 0 |
| #28 | 'Mammary Neoplasm, Human':ab,ti | 0 |
| #29 | 'Breast Carcinoma':ab,ti | 36,325 |
| #30 | 'Breast Carcinomas':ab,ti | 11,635 |
| #31 | 'Carcinoma, Breast':ab,ti | 1,487 |
| #32 | 'Carcinomas, Breast':ab,ti | 136 |
| #33 | 'Mammary Carcinoma, Human':ab,ti | 3 |
| #34 | 'Carcinoma, Human Mammary':ab,ti | 1 |
| #35 | 'Carcinomas, Human Mammary':ab,ti | 0 |
| #36 | 'Human Mammary Carcinomas':ab,ti | 173 |
| #37 | 'Mammary Carcinomas, Human':ab,ti | 0 |
| #38 | 'Human Mammary Carcinoma':ab,ti | 766 |
| #39 | #1 OR #2 OR #3 OR #4 OR #5 OR #6 OR #7 OR #8 OR #9 OR #10 OR #11 OR #12 OR #13 OR #14 OR #15 OR #16 OR #17 OR #18 OR #19 OR #20 OR #21 OR #22 OR #23 OR #24 OR #25 OR #26 OR #27 OR #28 OR #29 OR #30 OR #31 OR #32 OR #33 OR #34 OR #35 OR #36 OR #37 OR #38 | 721,438 |
| #40 | 'Dementia'/exp | 471,198 |
| #41 | 'Dementias':ab,ti | 10,479 |
| #42 | 'Amentia':ab,ti | 94 |
| #43 | 'Amentias':ab,ti | 2 |
| #44 | 'Senile Paranoid Dementia':ab,ti | 0 |
| #45 | 'Dementias, Senile Paranoid':ab,ti | 0 |
| #46 | 'Paranoid Dementia, Senile':ab,ti | 0 |
| #47 | 'Paranoid Dementias, Senile':ab,ti | 0 |
| #48 | 'Senile Paranoid Dementias':ab,ti | 0 |
| #49 | 'Familial Dementia':ab,ti | 131 |
| #50 | 'Dementia, Familial':ab,ti | 32 |
| #51 | 'Dementias, Familial':ab,ti | 3 |
| #52 | 'Familial Dementias':ab,ti | 26 |
| #53 | #40 OR #41 OR #42 OR #43 OR #44 OR #45 OR #46 OR #47 OR #48 OR #49 OR #50 OR #51 OR #52 | 472,167 |
| #54 | #39 AND #53 | 4335 |
