# Supplementary material for: Bidirectional association between breast cancer and dementia: a systematic review and meta-analysis of observational studies
Source: PeerJ. 2025 Jan 31;13:e18888. doi: 10.7717/peerj.18888 (PMC11789662; doi:10.7717/peerj.18888)
Supplement: Supplemental Information 9 [file peerj-13-18888-s009.docx]

**Supplemental Table 4** The excluded studies

| **Number** | **Author** | **Year** | **Title** | **Reason for excluded** |
| --- | --- | --- | --- | --- |
| 1 | hmad TA | 2024 | Prevalence of multimorbidity in survivors of 28 cancer sites: an English nationwide cross-sectional study | The full text does not meet eligibility criteria (no dementia) |
| 2 | Albers EAC | 2024 | The prevalence of mild cognitive impairment in breast cancer patients receiving chemotherapy according to the criteria of the National Institute on Aging-Alzheimer's Association (NIA-AA) | The full text does not meet the inclusion criteria (exposures were chemotherapy and control) |
| 3 | Li W | 2024 | Spatiotemporal Patterns of Hospitalizations Among Older Adults With Co-Presence of Cancer and Dementia in US Counties: 2013-2018 | The full text does not meet the eligibility criteria (co-presence of  cancer and dementia) |
| 4 | Li Y | 2024 | Association of Cancer History with Lifetime Risk of Dementia and Alzheimer's Disease | The full text does not meet the eligibility criteria (different outcome) |
| 5 | Mandelblatt J | 2024 | Alzheimer's disease-related biomarkers and cancer-related cognitive decline: the thinking and living with cancer study | The full text does not meet the eligibility criteria (different outcome) |
| 6 | Raver E | 2024 | Breast cancer screening among Medicare Advantage enrollees with dementia | The full text does not meet the eligibility criteria (different outcome) |
| 7 | Wang J | 2024 | Association of cancer history with structural brain aging markers of Alzheimer's disease and related dementias risk | The full text does not meet the eligibility criteria (different outcome) |
| 8 | Agapito I | 2023 | Neuropsychiatric complications and associated management in adolescent and young adult cancer survivors: An All of Us study | The full text does not meet the eligibility criteria (different outcome) |
| 9 | Kc M | 2023 | Relative Burden of Cancer and Noncancer Mortality Among Long-Term Survivors of Breast, Prostate, and Colorectal Cancer in the US | The full text does not meet the eligibility criteria |
| 10 | Schiaffino MK | 2023 | The disproportionate burden of Alzheimer's disease and related dementias (ADRD) in diverse older adults diagnosed with cancer | The full text does not meet the eligibility criteria (different outcome) |
| 11 | Schoenborn NL | 2023 | Breast and prostate cancer screening rates by cognitive status in US older adults | The full text does not meet the eligibility criteria (different outcome) |
| 12 | Stabellini N | 2023 | Racial disparities in breast cancer treatment patterns and treatment related adverse events | The full text does not meet the eligibility criteria (different outcome) |
| 13 | Weng X | 2023 | Alzheimer's disease and related dementias is a risk factor for lower utilization of breast cancer screening and unstaged cancer diagnosis: Observational study from SEER-Medicare 2004-2016 data | The full text does not meet the eligibility criteria (different outcome) |
| 14 | Xu WY | 2023 | Rural-urban disparities in preventive breast and cervical cancer screening among women with early-onset dementia | The full text does not meet the eligibility criteria (different outcome) |
| 15 | Du XL | 2022 | Risk of Developing Alzheimer's Disease and Related Dementias in Association with Cardiovascular Disease, Stroke, Hypertension, and Diabetes in a Large Cohort of Women with Breast Cancer and with up to 26 Years of Follow-Up | The full text does not meet the eligibility criteria (drug use) |
| 16 | González-Santos Á | 2022 | A Telehealth-Based Cognitive-Adaptive Training (e-OTCAT) to Prevent Cancer and Chemotherapy-Related Cognitive Impairment in Women with Breast Cancer: Protocol for a Randomized Controlled Trial | The full text does not meet the eligibility criteria |
| 17 | Hayes-Larson E | 2022 | The Role of Dementia Diagnostic Delay in the Inverse Cancer-Dementia Association | The full text does not meet the eligibility criteria (cancer) |
| 18 | Karanth SD | 2022 | Cancer diagnosis is associated with a lower burden of dementia and less Alzheimer's-type neuropathology | The full text does not meet the eligibility criteria (different outcome) |
| 19 | Li L | 2022 | Time trends in cancer and dementia related hospital admissions among Medicare fee-for-service population, 2013-2018 | The full text does not meet the eligibility criteria (different outcome) |
| 20 | Singhal J | 2022 | Public perceptions of predictive testing for rheumatoid arthritis compared to breast cancer and early-onset Alzheimer's disease: a qualitative study | The full text does not meet the eligibility criteria (different outcome) |
| 21 | Weng X | 2022 | A comparison of end-of-life care patterns between older patients with both cancer and Alzheimer's disease and related dementias versus those with only cancer | The full text does not meet the eligibility criteria (different outcome) |
| 22 | Chamberlain JD | 2021 | Investigating the association between cancer and the risk of dementia: Results from the Memento cohort | The full text does not meet the eligibility criteria (cancer) |
| 23 | Hsu WH | 2021 | Insufficient pain control for patients with cancer and dementia during terminal cancer stages | The full text does not meet the eligibility criteria (different outcome) |
| 24 | Megari K | 2021 | Neuropsychological functioning among patients with different types of cancer : Postchemotherapy cognitive impairment and implications for rehabilitation | The full text does not meet the eligibility criteria (different outcome) |
| 25 | Thompson MR | 2021 | Association of Endocrine Therapy and Dementia in Women with Breast Cancer | The full text does not meet the eligibility criteria (endocrine therapy) |
| 26 | Van Dyk K | 2021 | Cancer-Related Cognitive Impairment in Patients With a History of Breast Cancer | The full text does not meet the eligibility criteria (different outcome) |
| 27 | Katuwal S | 2021 | Causes of death among women with breast cancer: A follow-up study of 50 481 women with breast cancer in Finland | The full text does not meet the eligibility criteria (different outcome) |
| 28 | Martin C | 2021 | Treatment choices for older women with primary operable breast cancer and cognitive impairment: Results from a prospective, multicentre cohort study | The full text does not meet the eligibility criteria (different outcome) |
| 29 | Branigan GL | 2020 | Association Between Hormone-Modulating Breast Cancer Therapies and Incidence of Neurodegenerative Outcomes for Women With Breast Cancer | The full text does not meet the eligibility criteria (different outcome) |
| 30 | Fowler ME | 2020 | Progression of Alzheimer's Disease by Self-Reported Cancer History in the Alzheimer's Disease Neuroimaging Initiative | The full text does not meet the eligibility criteria (different outcome) |
| 31 | Mezencev R | 2020 | Risk of Alzheimer's Disease in Cancer Patients: Analysis of Mortality Data from the US SEER Population-Based Registries | The full text does not meet the eligibility criteria (different outcome) |
| 32 | Abdel-Rahman O | 2020 | Death from Alzheimer's disease among cancer survivors: a population-based study | The full text does not meet the eligibility criteria (different outcome) |
| 33 | Afifi AM | 2020 | Causes of death after breast cancer diagnosis: A US population-based analysis | The full text does not meet the eligibility criteria (different outcome) |
| 34 | Assis T | 2019 | Predictors of early seizure recurrence among elderly inpatients admitted to a tertiary center: A prospective cohort study | The full text does not meet the inclusion criteria (different outcome) |
| 35 | Bromley SE | 2019 | Risk of dementia among postmenopausal breast cancer survivors treated with aromatase inhibitors versus tamoxifen: a cohort study using primary care data from the UK | The full text does not meet the eligibility criteria (drug use) |
| 36 | Han KM | 2019 | Chronic medical conditions and metabolic syndrome as risk factors for incidence of major depressive disorder: A longitudinal study based on 4.7 million adults in South Korea | The full text does not meet the eligibility criteria (different outcome) |
| 37 | Haskins CB | 2019 | Impact of preexisting mental illness on breast cancer endocrine therapy adherence | The full text does not meet the eligibility criteria (therapy adherence) |
| 38 | Ojala K | 2019 | Surgical treatment and prognosis of breast cancer in elderly - A population-based study | The full text does not meet the eligibility criteria (different outcome) |
| 39 | Cacho-Díaz B | 2018 | Diagnosis of brain metastases in breast cancer patients resulting from neurological symptoms | The full text does not meet the eligibility criteria |
| 40 | Edwards BJ | 2018 | Neurocognitive deficits in older patients with cancer | The full text does not meet the eligibility criteria (different outcome) |
| 41 | Lin HF | 2018 | Tamoxifen usage correlates with increased risk of Parkinson's disease in older women with breast cancer: a case-control study in Taiwan | The full text does not meet the eligibility criteria (drug use) |
| 42 | Mirza AS | 2018 | Comorbidities, risk, and socioeconomic factors of uninsured cancer survivors | The full text does not meet the eligibility criteria (different outcome) |
| 43 | Pravettoni G | 2018 | Psychiatric, behavioral, and cognitive disorders in patients with extracranial cancers | The full text does not meet the eligibility criteria (extracranial cancers) |
| 44 | Smitherman AB | 2018 | Frailty and Comorbidities Among Survivors of Adolescent and Young Adult Cancer: A Cross-Sectional Examination of a Hospital-Based Survivorship Cohort | The full text does not meet the eligibility criteria (different outcome) |
| 45 | Ewertz M | 2018 | Influence of specific comorbidities on survival after early-stage breast cancer | The full text does not meet the eligibility criteria |
| 46 | Huang HK | 2017 | Do cancer patients with dementia receive less aggressive treatment in end-of-life care? A nationwide population-based cohort study | The full text does not meet the eligibility criteria (different outcome) |
| 47 | Kesler SR | 2017 | Probability of Alzheimer's disease in breast cancer survivors based on gray-matter structural network efficiency | The full text does not meet the eligibility criteria (different outcome) |
| 48 | Liao KF | 2017 | Nationwide Case-Control Study Examining the Association between Tamoxifen Use and Alzheimer's Disease in Aged Women with Breast Cancer in Taiwan | The full text does not meet the eligibility criteria (drug use) |
| 49 | Messmer MF | 2017 | I-SPY 2 Breast Cancer Trial as a Model for Innovation in Alzheimer Disease Therapies | The full text does not meet the eligibility criteria (different outcome) |
| 50 | Chang CM | 2016 | Adjusted Age-Adjusted Charlson Comorbidity Index Score as a Risk Measure of Perioperative Mortality before Cancer Surgery | The full text does not meet the eligibility criteria |
| 51 | Magnuson A | 2016 | Cognition and Cognitive Impairment in Older Adults with Cancer | The full text does not meet the eligibility criteria (cancer) |
| 52 | Lundquist TS | 2015 | Screening for Alzheimer's disease: inspiration and ideas from breast cancer strategies | The full text does not meet the eligibility criteria (review) |
| 53 | Pereira S | 2015 | Neurological complications of breast cancer: A prospective cohort study | The full text does not meet the eligibility criteria (different outcome) |
| 54 | Rahman MM | 2015 | Moving Beyond Cancer: Immediate Impact on-the Health-Related Quality of Life of Breast Cancer Patients After Mastectomy | The full text does not meet the eligibility criteria (different outcome) |
| 55 | Mandelblatt JS | 2014 | Cognitive impairment in older patients with breast cancer before systemic therapy: is there an interaction between cancer and comorbidity? | The full text does not meet the eligibility criteria (different outcome) |
| 56 | Ording AG | 2013 | Comorbidity and survival of Danish breast cancer patients from 2000-2011: a population-based cohort study | The full text does not meet the eligibility criteria (different outcome) |
| 57 | Ording AG | 2013 | Comorbid diseases interact with breast cancer to affect mortality in the first year after diagnosis--a Danish nationwide matched cohort study | The full text does not meet the eligibility criteria (different outcome) |
| 58 | Ording AG | 2013 | Null association between tamoxifen use and dementia in Danish breast cancer patients | The full text does not meet the eligibility criteria (drug use) |
| 59 | Jung SY | 2012 | Comorbidity as a mediator of survival disparity between younger and older women diagnosed with metastatic breast cancer | The full text does not meet the eligibility criteria (different outcome) |
| 60 | Tada Y | 2012 | Psychiatric disorders in cancer patients at a university hospital in Japan: descriptive analysis of 765 psychiatric referrals | The full text does not meet the eligibility criteria (different outcome) |
| 61 | Azoulay L | 2011 | The use of atypical antipsychotics and the risk of breast cancer | The full text does not meet the eligibility criteria (drug use) |
| 62 | Patnaik JL | 2011 | The influence of comorbidities on overall survival among older women diagnosed with breast cancer | The full text does not meet the eligibility criteria |
| 63 | Arrighi HM | 2010 | Prevalence and impact of dementia-related functional limitations in the United States, 2001 to 2005 | The full text does not meet the eligibility criteria (population) |
| 64 | Arrighi HM | 2010 | Lethality of Alzheimer disease and its impact on nursing home placement | The full text does not meet the eligibility criteria (different outcome) |
| 65 | Du XL | 2010 | Relationship between chemotherapy use and cognitive impairments in older women with breast cancer: findings from a large population-based cohort | The full text does not meet the eligibility criteria (chemotherapy) |
| 66 | Raji MA | 2009 | Risk of subsequent dementia diagnoses does not vary by types of adjuvant chemotherapy in older women with breast cancer | The full text does not meet the eligibility criteria (different outcome) |
| 67 | Heck JE | 2008 | Patterns of dementia diagnosis in surveillance, epidemiology, and end results breast cancer survivors who use chemotherapy | The full text does not meet the eligibility criteria (Chemotherapy) |
| 68 | Minisini AM | 2008 | Cognitive functions and elderly cancer patients receiving anticancer treatment: a prospective study | The full text does not meet the eligibility criteria (anticancer treatment) |
| 69 | Raji MA | 2008 | Effect of a dementia diagnosis on survival of older patients after a diagnosis of breast, colon, or prostate cancer: implications for cancer care | The full text does not meet the eligibility criteria (different outcome) |
| 70 | Gorin SS | 2005 | Treatment for breast cancer in patients with Alzheimer's disease | The full text does not meet the eligibility criteria (drug use) |
| 71 | Louwman WJ | 2005 | Less extensive treatment and inferior prognosis for breast cancer patient with comorbidity: a population-based study | The full text does not meet the eligibility criteria |
