# Supplementary material for: Bidirectional association between breast cancer and dementia: a systematic review and meta-analysis of observational studies
Source: PeerJ. 2025 Jan 31;13:e18888. doi: 10.7717/peerj.18888 (PMC11789662; doi:10.7717/peerj.18888)
Supplement: Supplemental Information 10 [file peerj-13-18888-s010.docx]

**Supplemental Table 5** Sensitivity analysis for breast cancer and the risk of dementia

|  |  | **OR** | **95% CI** | **I2/%** | ***P* value** |
| --- | --- | --- | --- | --- | --- |
|  | **Total** | 0.56 | 0.27-1.18 | 99.1 | 0.128 |
|  | **Excluded study** |  |  |  |  |
| 1 | Oh J 2023 | 0.80 | 0.57-1.12 | 93.8 | 0.193 |
| 2 | Roderburg C 2021 | 0.49 | 0.20-1.18 | 98.6 | 0.113 |
| 3 | Kurita GP 2017 | 0.59 | 0.26-1.32 | 99.2 | 0.201 |
| 4 | Musicco M 2013 | 0.55 | 0.20-1.46 | 99.2 | 0.230 |
| 5 | Jørgensen TL 2012 | 0.54 | 0.24-1.22 | 99.2 | 0.139 |
| 6 | Khan NF 2011 | 0.49 | 0.21-1.17 | 99.2 | 0.110 |
| 7 | Baxter NN 2009 | 0.54 | 0.23-1.27 | 99.2 | 0.159 |
