# Supplementary material for: Bidirectional association between breast cancer and dementia: a systematic review and meta-analysis of observational studies
Source: PeerJ. 2025 Jan 31;13:e18888. doi: 10.7717/peerj.18888 (PMC11789662; doi:10.7717/peerj.18888)
Supplement: Supplemental Information 11 [file peerj-13-18888-s011.docx]

**Supplemental Table 6** Sensitivity analysis for dementia and the risk of breast cancer

|  |  | **OR** | **95% CI** | **I2/%** | ***P* value** |
| --- | --- | --- | --- | --- | --- |
|  | **Total** | 0.79 | 0.51-1.22 | 94.5 | 0.290 |
|  | **Excluded study** |  |  |  |  |
| 1 | Ren RJ 2022 | 0.83 | 0.53-1.31 | 95.5 | 0.426 |
| 2 | Valentine D 2022 | 0.61 | 0.54-0.70 | 20.3 | 0.000 |
| 3 | Heun R 2013 | 0.77 | 0.47-1.25 | 95.6 | 0.293 |
| 4 | Musicco M 2013 | 0.91 | 0.65-1.27 | 67.0 | 0.570 |
| 5 | Ou SM 2013 | 0.77 | 0.47-1.26 | 95.5 | 0.302 |
| 6 | Attner B 2010 | 0.80 | 0.49-1.33 | 95.3 | 0.395 |
